# EMPOWER-support of patient empowerment by an intelligent self-management pathway for patients: study protocol

**DOI:** 10.1186/s12911-015-0142-x

**Published:** 2015-03-20

**Authors:** Sarah Mantwill, Maddalena Fiordelli, Ramona Ludolph, Peter J Schulz

**Affiliations:** Institute of Communication & Health, University of Lugano, Via Giuseppe Buffi 13, 6904 Lugano, Switzerland

**Keywords:** Empowerment, Self-management, Diabetes mellitus, mHealth, eHealth, ICT, Doctor patient communication, Study protocol

## Abstract

**Background:**

Diabetes education together with patient empowerment has shown to be key to effective self-management behavior. When delivered through information and communication technologies (ICT), this solution has shown to lead to better health outcomes. However, the potential of ICT and their integration into the healthcare environment have not yet been fully exploited. ICT should be in particular used to facilitate communication and information exchange between patient and healthcare providers. In addition, systems should include components facilitating behavior change using empowerment approaches such as goal-setting.

**Methods/Design:**

Funded by the European Commission (FP7-ICT-2011-288209) a web/mobile based platform (EMPOWER) has been developed, which aims at supporting self-management activities of diabetes patients and their treating physicians in Germany and Turkey. The platform semantically integrates multiple information sources, such as electronic and personal health records (EHR/PHR). Patients can register patterns of daily living, record blood glucose levels, design disease management plans and set long- and short-term goals. The project actively involves the treating physician, who has the possibility to set recommendations for the patient and to monitor his/her progress on the platform.

In the test-phase of EMPOWER, patients will be assigned to an intervention group and a control group. Data will be collected at baseline and three months after the intervention started. In addition, qualitative interviews will be conducted to collect extra information on usability and usefulness.

Outcome measures include amongst others the Problem Areas in Diabetes questionnaire (PAID), the Summary of Diabetes Self-Care Activities and scales evaluating doctor-patient interaction. Physiological parameters, such as physical activity or blood glucose levels will be collected via the platform. Further, log files and number of logins will serve as independent variables.

**Discussion:**

The interplay between multiple sources, including EHR, patients’ own registered information and physicians’ recommendations on one platform can have important practice implications. It might not only improve self-management activities in diabetes patients but it will also facilitate physician’s work, and ultimately the physician patient relationship.

**Trial registration:**

The trial has been registered with Deutsches Register Klinischer Studien (German register of clinical trials) under DRKS00007699 on January 30, 2015.

## Background

Diabetes, and in particular diabetes type 2, is one of the fastest growing chronic diseases in nowadays society [1]. Tight blood glucose control, dietary requirements and regular medication intake are only few of the things that a diabetes patient needs to manage in order to prevent long-term complications [2]. Given that continuity of care has to be guaranteed, this does not only pose many challenges for the patient but also for the treating physician who will need to support the patient’s management strategies.

Research has shown that diabetes education is central to effective self-management behavior, which in the long term can influence clinical [3] and psychological outcomes [4]. Lately, approaches have moved from purely educational interventions to those that empower patients based on the assumption that they are managers of their own health [5,6]. The term patient empowerment refers to a process that enables and facilitates behavior change. The key to empowerment does not necessarily lie in an increased compliance to what the doctor says or prescribes but rather in the opportunity to increase patients’ autonomy, and to improve their decision-making capacities [7].

Given the pervasiveness of Information and Communications Technologies (ICT) and their potential, eHealth and mHealth applications have become particularly popular for interventional approaches that aim at empowering patients. Studies in diabetes patients using web-based approaches have indeed shown to effect empowerment levels [8]. With regard to clinical outcomes, web-based interventions were able to reduce blood glucose levels [9] and hospitalization rates [10]. Even though effects found were in general rather small [11], web-based approaches, compared to traditional approaches, yield promising results [12]. Regarding the effectiveness of mobile applications, there is only few solid evidence on diabetes treatment so far, given that the field of mHealth is still very young [13,14]. However there are some recent encouraging findings that underscore the potential of mobile use in combination with web-based approaches for diabetes education purposes [11,15].

Most web-based interventions, so far, have not been comprehensively embedded into the healthcare environment surrounding the individual patient. Therefore, more systematical approaches are needed. Personal health records (PHR) that are linked to electronic health records (EHR) have shown to be a promising solution. PHRs allow patients to manage and share their health information using secure pathways with others who are authorized to see them [16]. Particularly in chronic disease management PHRs are of considerable value given that diabetes patients require constant care and regular follow-ups. In addition, time and resource constraints in physicians’ everyday practice make PHRs even more useful [17].

Studies that included PHRs and focused on diabetes were able to show that the use of PHRs linked to EHRs would increase for instance the adjustment of diabetes related medication [18] or improve blood glucose levels [19].

Goal-setting is one of the key components facilitating behavior change with the empowerment approach [7]. Goal-setting helps patients to act more independently by providing feedback on their self-management behavior thereby also sustaining motivation and increasing problem-solving skills and self-reflection. Goal-setting does not necessarily have to refer to purely clinical outcomes but also to psychological outcomes such as self-efficacy, which in the long run might influence the continuity or increase of an action set as a goal [20]. Even though goal-setting in collaboration with the treating physician is favorable, only few studies so far were able to show an active involvement of the treating physician. Studies showed that most physicians were reluctant to actively get involved, due to time and resource constraints. The usage of computer-based programs has shown to be helpful to, at least partly, circumvent these issues [20]. However, in spite of physicians’ reluctance, most of the interventions, including non-diabetes related studies, that used for example PHRs were mostly physician-oriented and did not take sufficiently the patients’ needs into account. For example, patients were only able to access their information via their physicians or treating hospitals. Besides, the general lack of additional information on diabetes treatment or any form of patient support proved those systems to be less attractive to patients [21].

In general, ICT should be used to facilitate communication and information exchange between patient and healthcare providers, which is fundamental to the motivation of the patient. ICT should not rely solely on information provision and data insertion but also contribute to an interactive exchange between the patient and his/her healthcare providers [22-24].

### Objective

The current study aims at increasing empowerment and self-management behaviors in a sample of diabetes patients type 1 and type 2 in Germany and Turkey by:

- Providing a web-based application, available also for mobile devices, that allows patients to regularly upload their personal health records in form of blood glucose measures or journal entries regarding physical activity, nutrition, etc.
- giving patients the opportunity to systematically define goals and track their improvements over the course of the intervention
- integrating physicians’ treatment goals and recommendations into the system
- systematically integrating the web-based application with electronic and personal health records

## Methods/Design

### The web-based application

**Figure 1:**
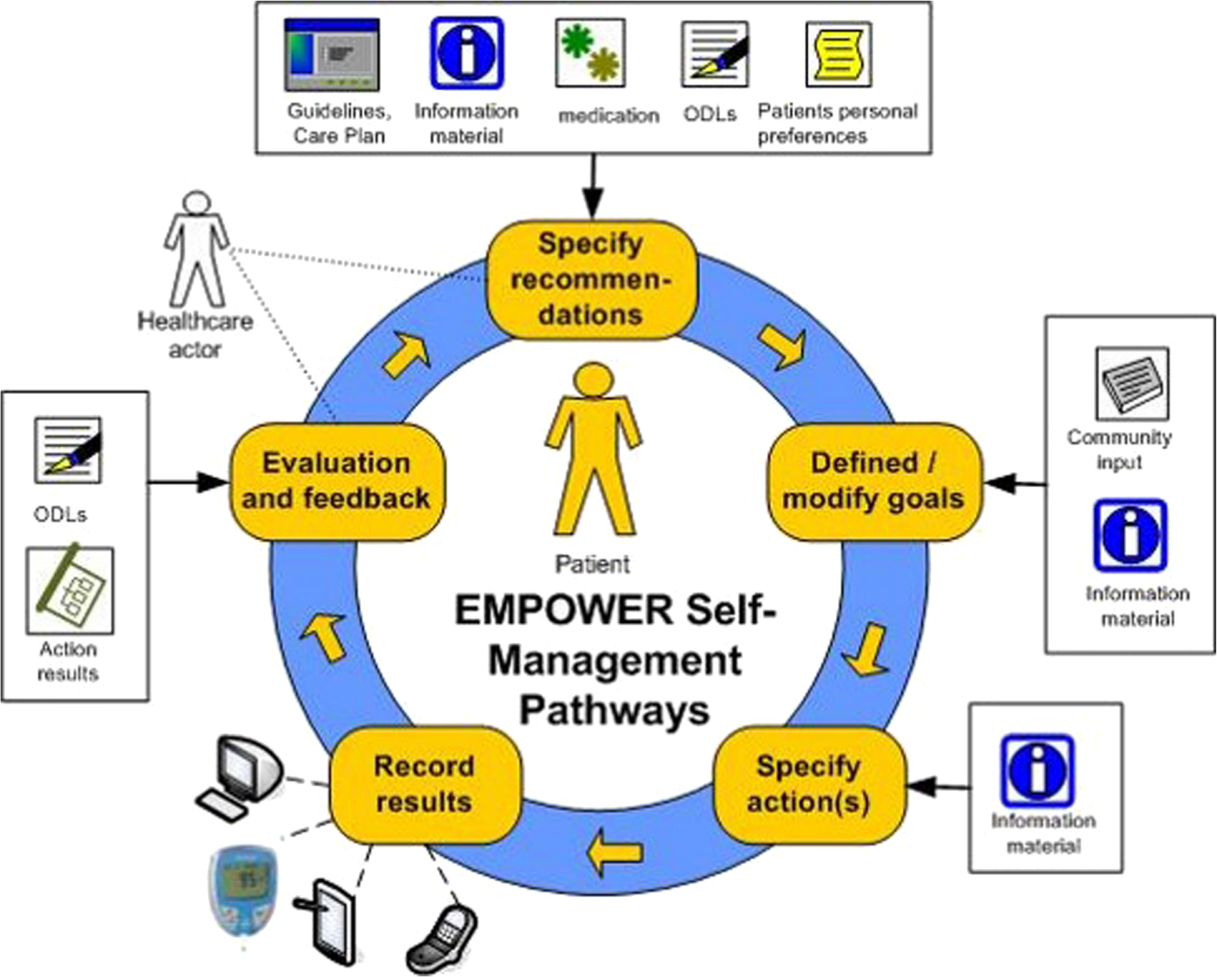
**EMPOWER cycle.**

The development of the application (EMPOWER system – Figure 1) encompasses four main objectives. The first one is to foster self-management with adaptive and secure patient pathways. The pathways are iterative and adaptable to the patients’ skills (access, competence and motivation), requirements and needs, which will be assessed by the system according to different maturity levels. Maturity levels – novice, advanced, and expert – refer here to the stage reached by the patient in learning to self-manage his/her diabetes management tasks.

A second objective is to support behavioral changes by integrating actions in patient’s daily life through personalized action plans. These actions plans will be based on recommendations from the treating physicians, personalized long-term goals, diabetes-relevant information material, and patients’ preferences. In addition, actions can be related to reminders in order to bring them timely into the patient’s mind. The third objective is to facilitate self-control by collecting patterns of daily living. Therefore, services for observations of daily living (ODLs) that allow patients to upload vital, physical (e.g. blood glucose levels) and mental parameters and physical and lifestyle activities are included in the system. The last objective is to include an open-source PHR system, which can be integrated into existing PHR or EHR systems. In turn, the EMPOWER system aims at integrating services from these other health applications. Semantic interoperability is supported on the basis of established standards (i.e. ISO/CEN13606 information models).

The EMPOWER system goes beyond already available diabetes self-management applications as it also involves the patients’ treating physician. During a routine consultation at the beginning of the intervention phase the collaborating physicians will specify treatment goals and recommendations regarding self-management goals together with the patient. This goal setting serves as the basis for the use of the EMPOWER system. Based on the patient’s consent the treating physician will be able to access the patient’s EMPOWER records and follow the patient’s self-management activities. Consequently, physicians will be able to detect possible causes for changes in the patient’s condition, such as fluctuating blood glucose levels. In addition, the physician will be able to discuss the patient’s goal pursuit process and to give advice on diabetes self-management.

**Figure 2:**
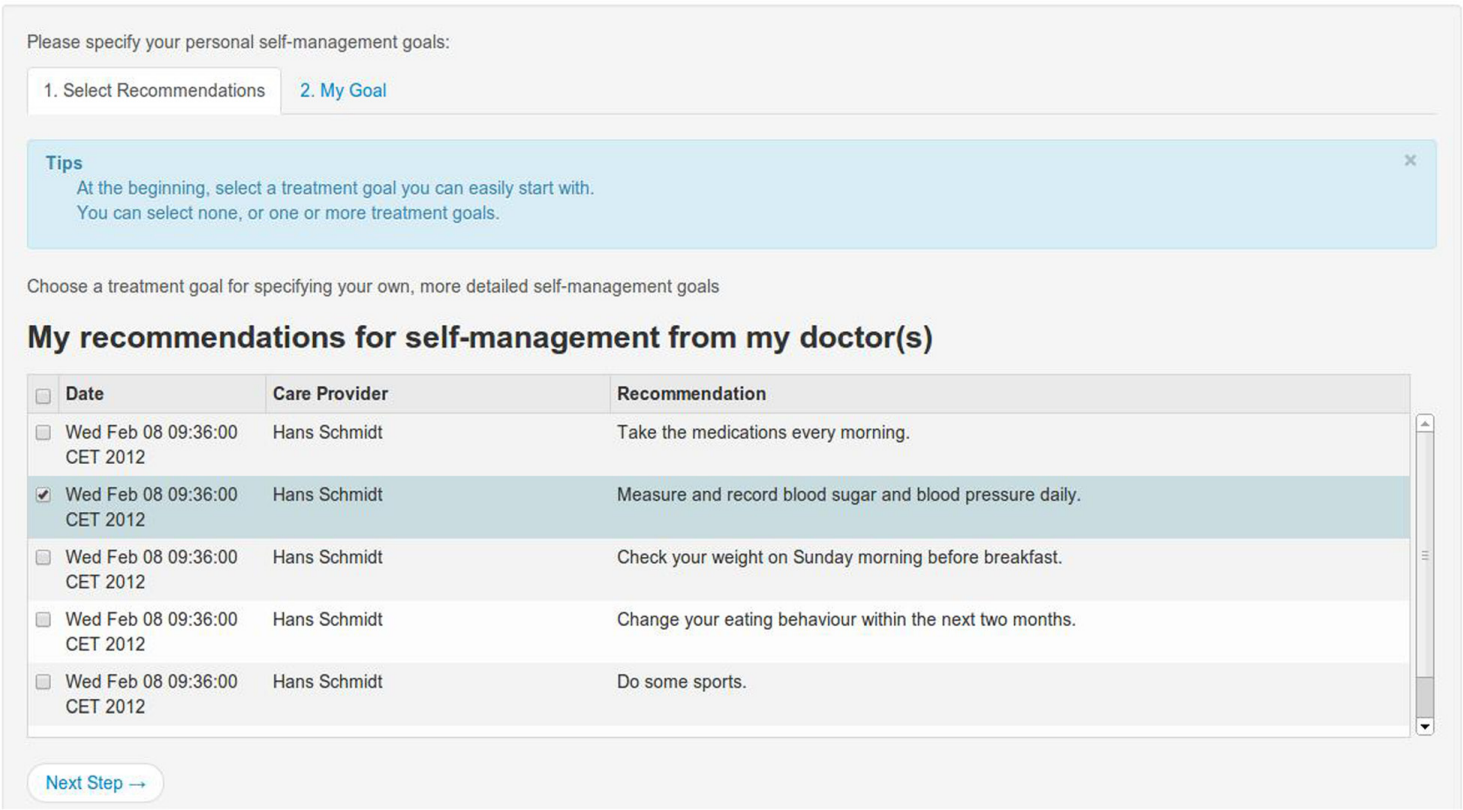
**Self-management portal: Physician’s recommendations to the patient.**

From a user experience perspective the EMPOWER system is divided into two main blocks. The first block targeting the treating physician is called *recommender engine*. The second block is the *self-management portal*, dedicated to the patients. The physician can monitor the patient’s health status through the recommender engine, which receives input from the patient’s Observations of Daily Living (ODLs). As a result, the physician can give the patient recommendations. A patient can check his/her physician’s recommendations, set his/her personal goals and plan his/her weekly activities in the self-management portal (Figure 2).

The patient can create journal entries during the week in order to get an overview of his/her progress, and use the review at the end of the week to evaluate the journal entries and to plan the activities of the forthcoming week. The patient decides on his/her own whether or not to grant access to his/her data to the physician by using the consent editor.

As a constant support, the patient can also access a section called patient information material, which offers disease related information and can be of help in planning goals and activities. The information material has been written in English for EMPOWER and then translated into German and Turkish. The material is based on existing and acknowledged information sources and educational material for diabetes patients. Since patients suffering from diabetes have different levels of knowledge, the material is designed to address different information needs: from basic information to more detailed, and from generic information to practical advice. For this purpose the Patient Information Material has been structured along three levels of content: Knowledge, Management, and Advice.

### Pretest of the EMPOWER Platform

The pilot application was pretested during a first validation phase in Germany and Turkey. Fifteen diabetes patients, thereof seven from Germany and eight from Turkey, first received a training on how to use the EMPOWER system and then used it for two months. Feedback on usability and usefulness was gathered quantitatively via standardized questionnaires as well as qualitatively through a roundtable discussion. The results of the first validation phase were then used to further develop the system and eliminate existing difficulties. Besides, the seven patients from Germany also participated in the pretest of the questionnaire on self-management that will be used as a baseline and post-intervention measure to assess the effectiveness of the EMPOWER system.

### Study design

The study is planned as a pre-post-design by administering questionnaires before and after the intervention. The aim is to recruit up to 70 participants at both study sites and then randomly assign them to either the intervention or control group.

After being recruited participants will be asked to give their informed consent. They will then be administered an online questionnaire containing various measures on self-management and socio-demographics to assess their baseline values before using the EMPOWER system. In order to create familiarity with the tool all participants will take part in an introductory training. There, patients will be taught how to download, use, and integrate the application into their daily life.

Participants are supposed to use the system regularly for a period of three months. They will be contacted via telephone on a weekly basis to gather feedback on usability, discuss potential problems concerning the tool and reduce the loss to follow-up. After the intervention has ended patients are again asked to fill in an online questionnaire containing the same measures as the pre-questionnaire plus questions on usability and usefulness of the tool.

### Ethical approval

The project was approved by the Bavarian State Office for Data Protection, Germany and by the Scientific Researches Evaluation Commission of the Ministry of Health of the Turkish Republic.

### Recruitment

It is planned to recruit overall 140 patients. The calculation of 140 participants (70 patients at each study site) is based on a previous study by Schulz and colleagues [25] in the field of web-based interventions. Eligible participants are between 18 and 65 years of age and suffer from diabetes type 1 or 2. Moreover, they need to have access to internet, own a computer or smart phone and be able to use the EMPOWER application.

In Germany, participants will be recruited via the practice network GOIN in Ingolstadt, Bavaria. The network comprises about 500 medical specialists and general practitioners who care for about 200.000 patients. The administrative staff from the practice network will be in charge of the recruitment of the patients and physicians for the main study. This will be done through personal contact with network members and advertising in practices and the network’s magazine.

Turkish participants will be recruited at the Hitit University Endocrinology Clinic of Turkey in Ankara. The Turkish Ministry of Health collaborates with two endocrinologists, two diabetes nurses, and one dietician at the University hospital who will recruit the participants among their patients, and also hired participants for the pretest.

### Outcome assessment

While the pre-questionnaire will only contain questions on self-management related constructs and socio-demographics, the post-questionnaire will also measure the perceived usability and usefulness of the application. Both questionnaires will be administered online and take about 20 to 30 minutes. Pre-post-comparisons will be facilitated by the random and thus anonymous assignment of a unique code for each participant that has to be entered when starting the survey. The questionnaire was created in English and then translated. Where available, already validated translations were used. For those scales that were only available in English, back and forward translations by native speakers of both languages were applied.

#### Patient information

Basic recordings about the patients comprise information such as socio-demographic descriptions, number of physician consultations during the intervention phase, or his/her experience with using similar technologies. Besides, the usage of EMPOWER will be recorded for each participant, including the patient’s number of logins to the system, the duration of use per visit, or the number of goals entered into the system. The records will show how often and intense the system was actually used by participants, and allow conclusions about the most useful features of the tool. Subjectively reported information from the patients will then be compared to objectively collected data.

#### Usability

Usability will be measured using the System Usability Scale by Brooke [26]. The scale was developed to assess the usability of interactive systems and is commonly used for this purpose. It consists of ten items that ask for a subjective assessment of the system, e.g. how easy it was to use it or how well integrated the various features of the system are. Both, reliability and validity of the scale have been proven [27]. Questions to assess the perceived usefulness of the EMPOWER system are based on the Technology Acceptance Model [28,29]. The model considers why technological systems are accepted or rejected by its users and how certain characteristics of the system determine this process. To measure the perceived usefulness of the EMPOWER system ten items from Davis’ [29] short screening scale on technology acceptance are used and were slightly adapted to the purposes of EMPOWER.

To assess whether the participating physicians perceive the EMPOWER system as useful in fostering patients’ self-management semi-structured interviews will be conducted. This will be done during the intervention phase via short telephone interviews to gather current feedback on the tool and more detailed through face-to-face interviews after the intervention.

#### Empowerment

Patients’ empowerment will be measured using Spreitzer’s Empowerment Scale [30] and The Problem Areas in Diabetes (PAID) [31]. Spreitzer’s Empowerment Scale consists of twelve statements related to the patient’s perceived importance, control, management, and autonomy concerning his/her diabetes. The PAID asks the patient whether he/she perceives twenty aspects, such as being limited concerning nutrition or not having treatment goals, as current problems.

#### Self-management activities

The impact of patient empowerment on self-management activities through the use of the EMPOWER system will be assessed by measuring the patients’ pre- and post-intervention health status, diabetes self-care, health literacy, doctor-patient communication, and empowerment. To assess an overall effect of the application on the health status as perceived by the patient, a self-reported, one item measure will be used. Patients will be asked to rate their health independently from their diabetes to avoid negatively biased answers due to the chronic condition. Diabetes self-care will also be measured subjectively, using the SDSCA as developed by Toobert and Glasgow [32] and revised by Toobert and colleagues [33]. The scale focuses on the patients’ self-care across various dimensions such as diet, exercise, blood sugar testing, foot care, and smoking. The questions refer to the last seven days of a participant’s routine diabetes self-care and therefore represent a comprehensive insight into the patient’s self-management that will be completed by the objective data stemming from the user tracking.

#### Health literacy

As an adequate level of heath literacy is paramount for being an effective self-manager [34] possible changes in health literacy due to the use of the EMPOWER system are assessed. Therefore, the Newest Vital Sign (NVS) [35], an objective test of functional health literacy in form of a nutrition label will be employed. Further, a subjective measure consisting of three items related to the perceived ability to understand written medical information, namely the Chew Items [36,37], will be used. In order to gain insight in the patients’ knowledge specifically about diabetes a range of knowledge items will be asked. Doctor-patient communication will be measured with scales from Kaplan [38] and Heisler [39]. While the first one assesses a physician’s decision making style concerning the patient’s involvement in treatment decisions, the latter one focuses on the provider communication. It thus considers the patient’s satisfaction with his/her doctor’s communication concerning disease and treatment.

### Data analysis

Data will be analyzed using the statistics software package SPSS, version 21, from IBM. To detect possible changes and their predictors’ descriptive statistics and regression analyses will be applied. In order to compare pre- and post-results as well as control and intervention group dependent and independent t-tests will be calculated.

## Discussion

The interplay between multiple sources, including EHRs, patients’ own registered information and treating physicians’ recommendations on one platform can have important implications for general practice. By actively integrating these features in one comprehensive platform the EMPOWER system may not only improve self-management activities in diabetes patients but will also facilitate physician’s work by having all important information readily available on one platform. In addition, the system may help improve the relationship between doctor and patient and foster a more systematic discussion with the patient on further disease management strategies and potential intervention points.

### Limitations

Limitations are prone to occur with regard to patient recruitment. The intervention will have to foresee potential dropouts due to the length of the intervention and potential research overload on the side of the patient.

In addition, physicians are asked to be actively involved in the program but time constraints and resource limitations are likely to impact their involvement. Nevertheless, with regard to patient retention rates physicians are of crucial importance in order to promote and sustain the intervention.

Lastly, given the fairly short amount of time of three months, behavioral changes may not be easily detectable, therefore regular use of the EMPOWER system has to be made sure in form of reminders and regular updates regarding changes of the system.

## Abbreviations

EHR: Electronic health records
ICT: Information and communication technologies
ODL: Observations of daily living
PHR: Personal health records
